# Approximate Priority Hybrid 3DNoC Buffered-Bufferless Router

**DOI:** 10.3390/mi14020335

**Published:** 2023-01-28

**Authors:** Savvas Savva, Konstantinos Tatas, Costas Kyriacou

**Affiliations:** 1 Department of Electrical Engineering, Computer Engineering and Informatics, Frederick University, 1036 Nicosia, Cyprus; 2 Frederick Research Center and Department of Electrical Engineering, Computer Engineering and Informatics, Frederick University, 1036 Nicosia, Cyprus

**Keywords:** 3D Networks-on-Chip, NoC, bufferless routing, approximate computing

## Abstract

This paper introduces a novel 3D NoC router that combines buffered and bufferless routing with approximate priority comparison when deflecting flits. Our proposal is a modification of an asymmetrical router that is buffered in the z dimension ports and bufferless in the x and y dimension ports. Flits that request output ports in the x and y dimensions are granted or deflected based on approximate, instead of accurate, priority comparison. Experimental results show that the proposed router, in addition to effectively combining the advantages of both buffered and bufferless routers, achieves additional performance and area gains due to the reduced logic required for approximate priority comparison in flit deflections. Experimental results using synthetic and realistic traffic show that the proposed router begins to saturate at a significantly higher injection rate than a bufferless router, but at a slightly lower injection rate than when using accurate priority comparison. Furthermore, the proposed router achieves higher clock frequencies and a reduced area compared to bufferles routers due to the simpler permutation network. The increased routing efficiency is shown to also translate to energy gains.

## 1. Introduction

As power and heat density limited the growth of clock frequencies compared to the prediction of Moore’s law, the dominant design paradigm for processors became the multicore architecture. At the same time, this exchanged the power density problem with the core communication challenge. Networks-on-Chip was proposed as a scalable solution providing the communication bandwidth required by multi and many-core architectures with acceptable area and power consumption [1]. The advance of 3D integration provided an additional incentive, since 3D integration combined with NoCs led to the emergence of 3D NoC architectures [1]. A key element in the NoC is the router, which is responsible for forwarding packets through the network, since it has strict requirements for performance and reliability in the aggressive scaling of CMOS technology [2].

**Figure 1:**
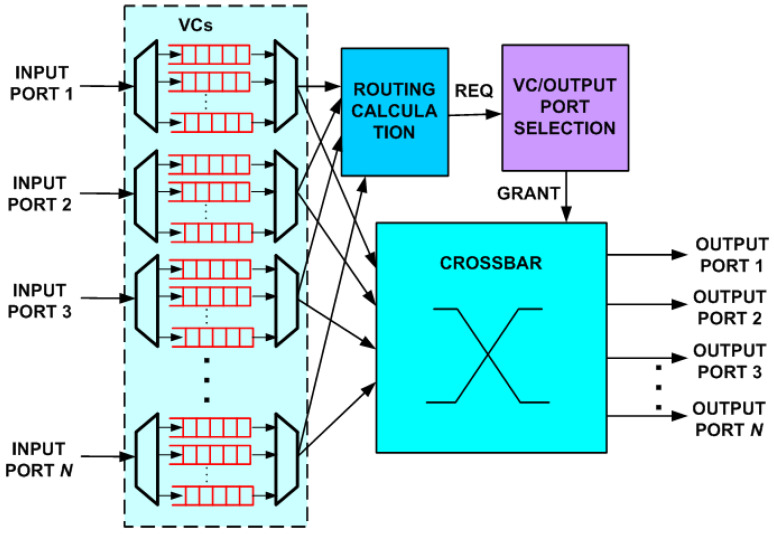
Generic Buffered NoC *N*-port Router Architecture with virtual channels.

Original NoC routers were on-chip implementations of interconnection network routers with little regard for the unique conditions and stringent requirements imposed by the on-chip environment [1,3]. The block diagram of such a router is shown in Figure 1. Typical router parameters that depend on network conditions and influence performance, area and power consumption, are flit size, flits per buffer and number of virtual channels. A routing table or a simple logic-based routing technique is used for routing calculation. Typically, each input port has its own private buffers with a number of Virtual Channels (VCs) used to prevent deadlock. The number of input and output ports depends on the topology. A common instance of the router in Figure 1 is a five-port version for 2D mesh and torus topologies.

Later studies considered the distinctive features of the on-chip environment driven by Moore’s law, which led to efforts to optimize the buffers in the router, since they were identified as the power and performance bottleneck. On the other hand, unlike the off-chip environment, wide flits are easy to implement on-chip, leading to higher parallelization.

When buffering is insufficient, NoCs can incorporate hot-potato routing or deflection routing: deflecting flits when buffered slots are unavailable. An extension of this was the even more radical suggestion of completely bufferless routing; in other words, forwarding flits to either the desired port or deflecting them, but never locally storing them in the router. This approach trades off routing efficiency (since some flits follow non-minimal paths due to deflections) for router area and power consumption due to the elimination of buffers. The later development of 3D integration led to adapting the routers originally proposed for 2D NoCs to 3D topologies.

## 2. Background and Related Work

In this section, we briefly discuss the evolution of NoC routers in terms of buffer organization and the emergence of bufferless routing as a viable router architecture, as well as the challenges imposed on router design by 3D integration.

### 2.1. Background

Buffering is a key component of router design due to its impact on router power consumption and area. Various approaches to buffer organization have been proposed, which can be classified as belonging to one of the following strategies [1]:

- Static approaches [4,5]: In this approach, the buffer sizes are static. Either all routers are identical in buffer size, or the optimal buffer organization is determined at design time through design space exploration, usually for a specific application(s).
- Run-time buffer allocation of a shared centralized buffer [6,7,8,9,10]: Typically, a centralized or shared buffer is dynamically allocated to VCs according to real-time traffic requirements. This approach provides adaptivity, unlike the previous one.
- Buffer bypassing [11]: This approach recognizes that buffers often become a performance bottleneck and seeks to bypass router buffers as much as possible.
- Deflection routing [12,13]: These approaches seek to reduce buffer size or completely eliminate buffers by misrouting (deflecting) incoming packets.

The last approach was introduced in [12], making the claim that completely bufferless routing demonstrates significant power gains compared to buffered routing, at a reasonable trade-off of some performance, that would be negligible at low injection rates. However, buffered router networks outperform bufferless ones at high injection rates as they exhibit higher network saturation points than buffered ones. In [13,14], the authors made cases for and against bufferless routing, respectively. It is precisely these trade-offs that have motivated the authors to search for a “middle” ground between buffered and bufferless routing that would provide a new solution.

One way to define the network saturation point is the following:

**Definition** **1.** *The injection rate for which the network latency is double the zero-load latency of the same network is the saturation injection rate or saturation threshold of the network.*

**Definition** **2.** *The value of network latency equal to double the zero load latency is the saturation latency.*

Unlike buffered NoCs, saturation in bufferless NoCs is due to a large number of flit deflections, not overrun buffers; 3D integration imposes additional challenges to NoC design, since packets must now also travel in the third dimension. This has led to the extension of both buffered and bufferless routers to the third dimension by adding two additional ports.

### 2.2. Related Work

The introduction of 3D NoC topologies added the requirement for efficiently supporting the third dimension to existing router designs. Extending the common 2D five-port router used in 2D mesh and torus topologies to seven ports by adding two additional ports was the reasonable approach [1]. However, this extension is costly in terms of chip area because the crossbar area quadratically scales with the number of ports [1]. Therefore, the 3D mesh router 7 × 7 crossbar occupies approximately double the area of the 2D mesh router 5 × 5 crossbar.

**Figure 2:**
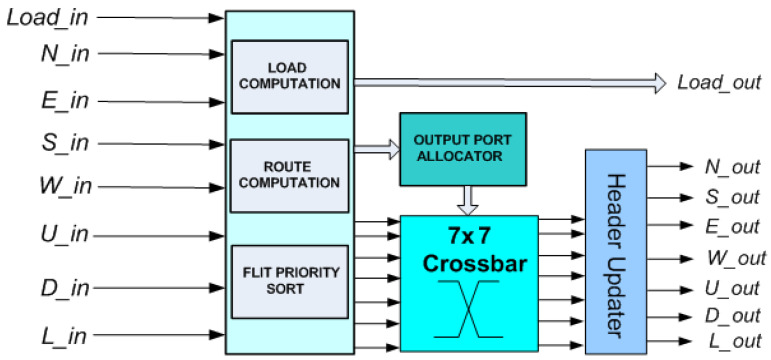
Baseline seven-port router for 3D bufferless NoCs (3DBASE).

The above holds for both buffered and bufferless crossbar-based routers, such as 3DBASE (Figure 2). Additionally, in order to avoid deadlock, the baseline bufferless router sorts incoming flits by priority, so that the flit with the highest priority is always assigned to its preferred port. The usual priority metric is the packet age. This ensures freedom from the livelock, since packets that have been in the network for a long time will have priority over “younger” packets. It also requires the packet age field to be updated (incremented) by every router in the routing path.

In [15], a bufferless router with dual ejection ports for 2D and 3D NoC was proposed. It is a one-cycle bufferless router that replaces the Flit Ejector module and a MUX module in a baseline bufferless router with a simple MUX module to achieve higher performance. This approach reduces the costly deflections that occur when two flits need to be ejected at the same time, and therefore, one is deflected back into the network. However, this approach does not improve the latency in the case of flits that are deflected before reaching the last router in their path.

**Figure 3:**
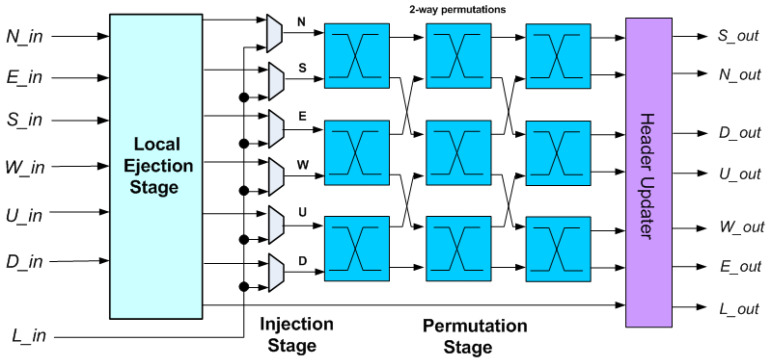
Bufferless router proposed in [16] (3DPERM).

In order to overcome the limitations of the crossbar, a single-cycle 3D bufferless router called 3DPERM with a three-stage permutation network that permutes packets based on packet age was introduced in [16]. The three-stage permutation network of 3DPERM is composed of nine permuter blocks, as shown in Figure 3. 3DPERM requires less area compared to 3DBASE, but features a lower saturation point due to the elimination of the load computation and priority sort, as well as higher end-to-end latency in cycles, particularly after crossing the saturation point. However, 3DPERM features lower end-to-end latency in nanoseconds at the low injection rates, due to the shorter critical path, and therefore, features a higher operating frequency [16]. Essentially, the permutation network approach trades-off some routing efficiency and lower saturation point for higher performance below the saturation point. Note that a permutation network-based 3D router uses nine permutation blocks instead of four for a 2D one, again requiring more than double the area.

We implemented 3DPERM in Nangate 45 nm technology and analyzed its area and critical path, as shown in Table 1 and Table 2.

**Figure 4:**
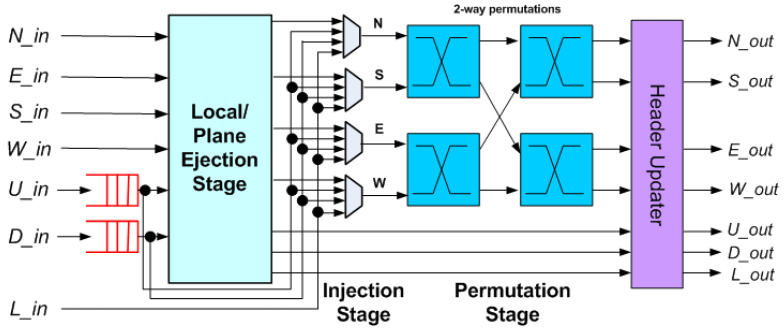
3DBUFFBLESS architecture.

In our previous work [17], an asymmetrical buffered-bufferless hybrid router for 3D NoC architectures called 3DBUFFBLESS (Figure 4) was proposed. The router was evaluated through simulation in terms of latency in cycles and number of hops, and through hardware implementation; ASIC synthesis resulted in a 45 nm technology in order to demonstrate that the router was a viable alternative to fully buffered and completely bufferless routers. Comparisons with 3DBASE and 3DPERM bufferless routers showed that 3DBUFFBLESS improves the network saturation point and achieves significantly higher performance at modest area and power costs.

A different approach to asymetrical buffered/bufferless routing was demonstrated in [18,19]. In [18], an asymmetric routing approach in bufferless 3D NoC proposed using interleaved edge routers to increase NoC performance. The approach in [19] was demonstrated in a 2D network on FPGA. Both approaches featured asymetryat the network level, combining buffered with bufferless routers in the same network, while our work featured a hybrid buffered/bufferless router.

In [20], we presented a different approach for improving a permutation network-based bufferless router: permuting and ejecting flits based on the approximate instead of accurate comparison of the priority metric (packet age). By only comparing a subset of the bits in the packet age, flits were classified as old, medium and young, giving priority to one age class over another while selecting pseudo-randomly between two packets in the same age class. Experimental results showed that this approach, while simplifying the calculation of packet priority, still ensures that older packets have priority over younger ones. The simplified permutation logic led to a higher operating frequency and reduced area at the cost of slightly reduced routing efficiency, since more packets are misrouted.

Since then, approximate bufferless routing has been used in ABNOC, an approximate bufferless NoC, proposed in [21], that uses an approximate allocation mechanism and a packet approximation method to decrease the packet retransmissions and network conflicts. Evaluation results, under synthetic traffic, guarantee retransmission and latency reduction. However, the design was evaluated and an improvement in bandwidth was demonstrated compared to previous work [22].

## 3. Proposed Hybrid Approximate Priority Router Design

The above innovations presented in [17,20] are essentially orthogonal, a fact that naturally leads to combining the two into a single router, exploiting the advantages of both. The proposed router, named 3DHYAP for a 3D Hybrid Approximate Priority Router, is based on the design in Figure 4, augmented with the approximate priority comparison of [20]. The proposed router combines the low cost of bufferless routing, augmented with approximate priority comparison, with the increased routing efficiency of partially buffered routing. In order to minimize hops on the vertical links (TSVs), the proposed router, like 3DBUFFBLESS, features buffering in the up and down ports and no buffering in the ports lying on the same plane. This allows a flit to quickly traverse the chip layers without being deflected, while minimizing the router buffering to only two of the total seven ports.

The reason the z direction was selected for buffering was the asymmetry imposed in the z dimension at the silicon level by the vertical links. Two of the most common ways for implementing vertical links, namely through silicon via (TSV) [23] and near-field inductive coupling (NFIC), impose an area overhead and also introduce errors and failures, reducing yield. Therefore, designers often choose to reduce the number of vertical links, leading to irregular, partially connected 3D architectures [24] (not a full mesh). This includes the wireless vertical link 3D NoCs in [25,26]. Of course, essentially, the design is dimension-agnostic, and the final floorplanning and layout connecting routers together selects which dimension the buffering is connected to. Any dimension can be selected for buffering without any HDL source code modification.

Since a packet may traverse the z dimension of the network, similarly to wormhole routing, but may have its flits deflected to different directions when moving in the xy plane, bufferless routing mechanisms such as livelock prevention and flit reordering are still required. However, with no horizontal buffer connections, deadlock is not an issue in a network composed of 3DHYAP routers, because buffered cyclic paths cannot be formed. Therefore, virtual channels are not required, simplifying the design of 3DHYAP.

The bufferless part of the router is based on a two-stage permutation network, as shown in Figure 4, but the selector and permutation blocks use approximate priority comparison.

### 3.1. Quantitative Analysis

The proposed design can be quantitatively analyzed using simple, back-of-the-envelope calculations and the experimental results in Table 1 and Table 2. When it comes to performance, we expect the permutation network delay to be reduced by one third because it will have two stages instead of three. Since the contribution of the permutation network to the critical path is 71% in 3DPERM, according to Ahmdal’s law we expect an improvement of:

(1) $$tt_{3\mathrm{DHYAPDHYAP}}=\frac{2}{3}\times 71\%\times tt_{3\mathrm{DPERMDPERM}}+29\%\times tt_{3\mathrm{DPERMDPERM}}=76.33\%\times tt_{3\mathrm{DPERMDPERM}}$$

In other words, we expect a reduction in delay and an improvement in clock frequency by 23.66% by the reduction in permutation stages. We expect an additional improvement by reducing the complexity of each permutation block by using approximate priority comparison.

In terms of area, the area gains obtained by having four instead of nine permutation blocks will be partly offset by the increased size of the injection/ejection stage, which now will have approximately three times the area of the original. Therefore:

(2) $$AA_{3\mathrm{DHYAPDHYAP}}=\left(3\times 9.1\%+\frac{4}{9}\times 75\%+19\%\right)\times AA_{3\mathrm{DPERMDPERM}}=80.5\%\times {{AAA}_{3\mathrm{DPERMDPERM}}}_{\mathrm{DPERM}}$$

Therefore, we expect an area reduction of approximately 20%. We should have additional area gains from the approximate priority comparison that will be partly offset by the addition of buffers. Clearly, the final area gains will depend on buffer size, and we explore this in the hardware evaluation section.

Integrating a buffered and a bufferless router in a seamless way proved challenging in the case of 3DBUFBLESS, and adding approximate priority comparison requires additional appropriate modifications made to the buffered and bufferless baseline router design, as discussed below.

### 3.2. General Considerations

Similar to 3DBUFBLESS, 3DHYAP features two additional injection and ejection ports on the router datapath in addition to the local port. Essentially, the up and down ports (buffered ports) are similar to the local port. Therefore, the ejection/injection stage is modified to contain three ports instead of one. Consequently, up to three additional flits may be injected into the bufferless part of the router at the same time (from the U_in, D_in and L_in ports). For this purpose, there are three stall signals, one for each port. This indicates the following possible conditions:

- No deflection output ports are available: This condition occurs when there are already four incoming flits from the bufferless input ports and neither is to be ejected. Then, since all incoming flits from bufferless ports must be assigned an output port, no buffered flits can be injected until the next clock cycle.
- There is one available deflection output port: This occurs when there are three requests from incoming flits arriving from bufferless ports that are not ejected. In this case, we make the following distinctions: if one of the ports requesting injection is the local port, then it is granted and the other(s) port (up or down) is/are stalled. This is meant to ensure that flits are injected to the network as soon as possible. If the only ports requesting injection are the up and down ports, we decide between the two flits based on their age.
- There are two available deflection output ports: In other words, there are two requests from incoming flits that are not ejected. In this case, up to two flits can be injected. In the case of all three injection ports making a request, the one flit granted is the local port’s, and the other one is selected from the other two based on age.
- There are two or more flits to be ejected, with at least one from a bufferless input port: In this case, a flit from a bufferless port is selected for ejection, so as not to be deflected. If there is more than one, the oldest is selected.
- There are two flits to be ejected, both from the buffered ports: One flit is selected according to age, the other remains buffered until the next cycle.

### 3.3. Priority Classes and Rules

The above considerations are formalized in the following eight ejection/injection rules, which resolve priority of the various types of incoming flits for ejection and injection. The first six also apply to 3DBUFFBLESS, with the last two added because of the approximate priority comparison:

1. If two or more flits incoming from the bufferless ports request ejection to a local, up or down port, the flit with the highest priority wins, while the rest are injected into the permutation network.
2. If an incoming flit from a bufferless input port competes for an ejection port with an incoming flit from a buffered input port, it is ejected while the other remains buffered, and waits for the next cycle.
3. If two incoming flits from buffered ports compete for an ejection port, one is selected according to rules 7 and 8, the other remains buffered.
4. Incoming flits from bufferless ports win over flits trying to inject from the local and up/down ports, which remain buffered.
5. If two or more injection ports are competing, then the local port wins over the up/down ports.
6. If the up and down ports are competing for injection, then rules 7 and 8 apply.
7. A flit in an “older” age class has priority over a younger flit.
8. Two flits belonging to the same age class are permuted in a permutation block, or one is pseudorandomly selected in a selector block.

Therefore, we distinguish between two priority classes: priority of an input port over another input port, and of a flit over another flit.

The priority of ports is resolved first and, if it is equal, then the priority of the individual flits is taken into account. We distinguish between three classes of ports: bufferless input ports (N, S, E, W), buffered input ports (U, D) and the local port (L). For injection, the set of input ports competing are (L, U, D), while, for ejection, the set of ports competing are (N, S, E, W, U, D). Then, the priority of the port classes is as follows:

Priority between flits is resolved using the following rules:
Ejection: Bufferless ports have higher priority than buffered ones
Injection: The local port has higher priority than the up and down ports

The rationale behind the ejection priority rule is to prevent flits from bufferless ports from being deflected when reaching their destination or wish to change layer, while flits from buffered ports can simply wait for the next cycle.

The ejection rule enforces “hot-potato” routing for flits that cannot be buffered, while the injection rule ensures that flits are quickly injected to the network and not “trapped” at the source for long. Since there are equal bufferless input and output ports, incoming flits from bufferless ports cannot be dropped. The only case when flits may be dropped is in the case of buffer overrun in the buffered ports.

### 3.4. Buffered Port Design

**Figure 5:**
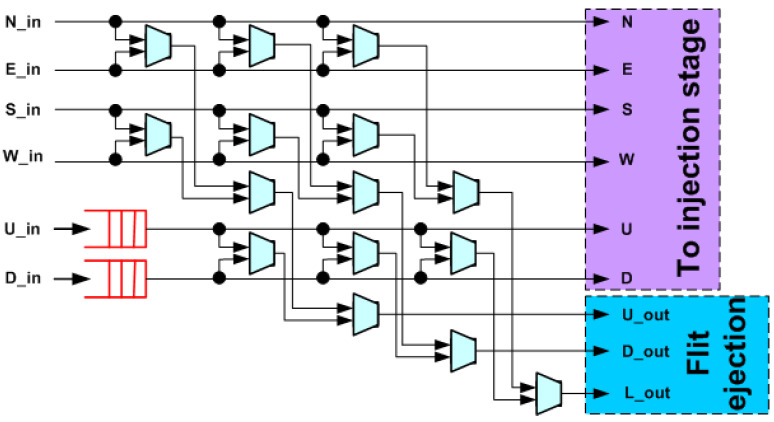
3DBUFFBLESS and 3DHYAP injection/ejection block.

The injection/ejection stage is shown in Figure 5. There are two similar ejection ports for the up and down directions, which have the local port as input. This incurs no significant performance penalty since the three ejection paths operate in parallel.

### 3.5. Injection/Ejection Stage

**Figure 6:**
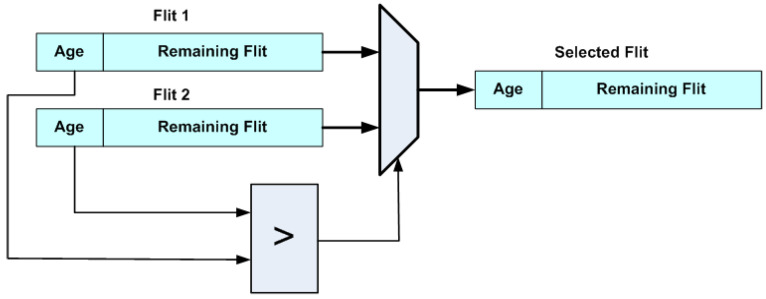
3DHYAP selector block.

The ejection stage selects, at most, one flit to be assigned to each of the ejection ports, U_out (up direction), D_out (down direction) and L_out (local port), based on priority while forwarding the remaining flits to the injection stage. As shown in Figure 5, the ejection stage is composed of three trees of flit selector blocks. Each selector accepts two flits as inputs and outputs the one with the highest priority, as shown in Figure 6. This way, the flit with the highest priority that has reached its destination is selected for ejection to the L_out port, while the flit with the highest priority that wishes to exit to the upper layer is forwarded to U_out, and likewise to the D_out for the lower layer. The remaining flits are either forwarded to the injection stage or remain buffered.

### 3.6. Approximate Priority Permutation Network

**Figure 7:**
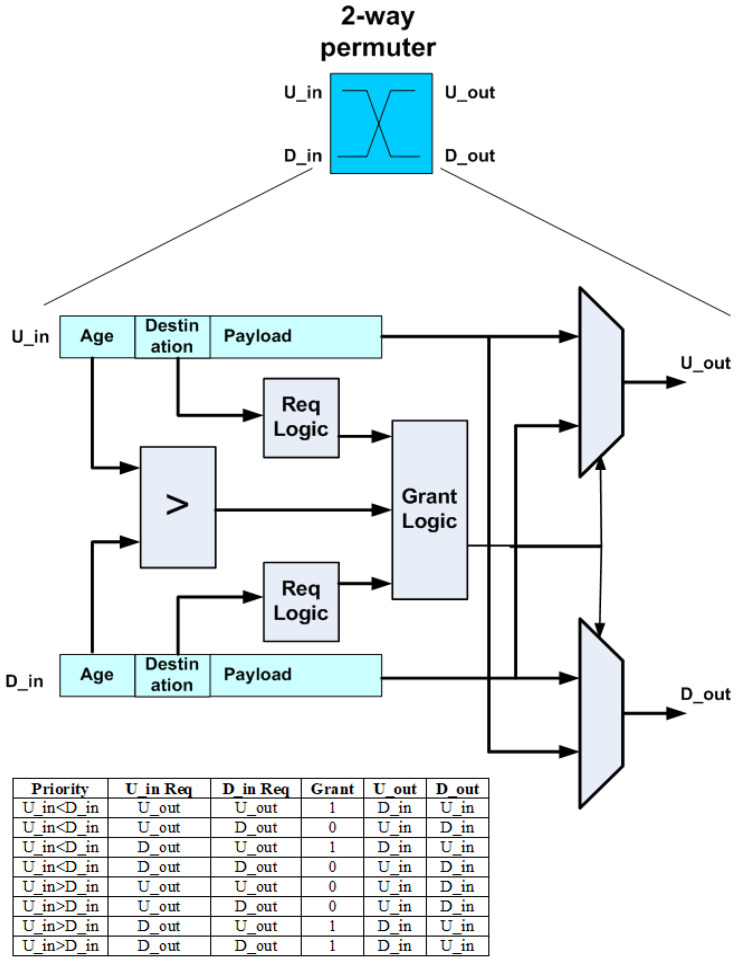
3DHYAP permutation block.

A permutation block is similar to a selector block, but requires two multiplexers instead of one as it permutes two flits, as shown in Figure 7. If the incoming flits are requesting different outputs, they can both be granted their request. However, when they both request the same output, either U_out or D_out, the one with the highest age field value wins, and the other is deflected to the other permuter output.

3DHYAP adopts the approximate comparison logic of 3DAPBLESS [20], where the magnitude comparator is replaced with simpler logic that compares a subset of the bits in the age field of the competing flits. By only comparing the most significant bits, 3DHYAP essentially separates the flits as belonging to crisp “age classes”.

When competing flits belong to the same age class, they are pseudorandomly permuted. We use a single 16-bit PSRNG, with one bit feeding each of the permutation and ejection blocks. We demonstrate two approximations, as shown in Table 3, using the two most significant bits, and using only a single most significant bit. Using two bits separates flit ages into four classes, while using only one separates them into two. Essentially a flit with an MSB of 1 in the age field is classified as “old”, while a flit with an MSB of 0 is classified as “young”.

**Figure 8:**
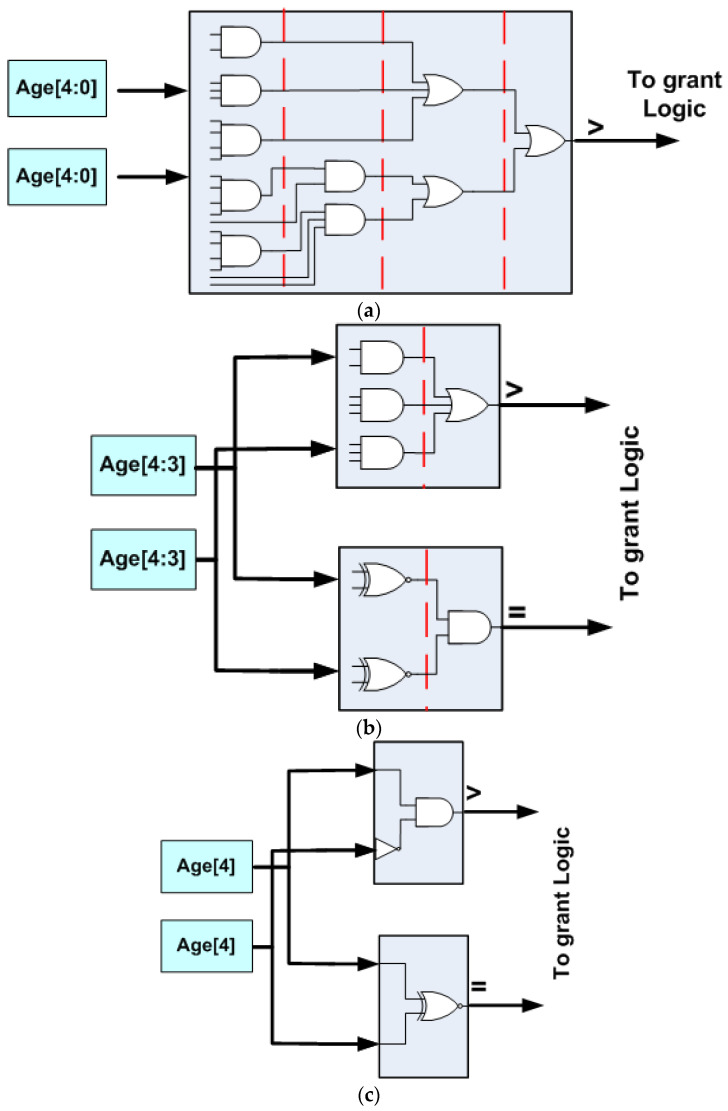
Reduced levels of logic using approximate comparison. (**a**) Full comparison, (**b**) 3DHYAP and (**c**) 3DHYAP_lite.

In our evaluation section, we consider a 4 × 4 × 3 mesh NoC. In this case, the maximum internode distance is eight hops. Since a reasonable age field would include at least double that number, we use five bits in our design (Figure 8a). Similar to [20], we have experimented with two versions of 3DHYAP, one using the two most significant bits of the age field and one using only one, which we term 3DHYAP_lite.

We next attempt to estimate the additional performance improvement achieved by the approximate priority comparison. Figure 8a shows the magnitude comparator for a 5-bit age field (inverters not shown). As can be seen, after breaking down the logic to four-input logic gates at most, four levels of logic are required. In Figure 8b, the equivalent circuit with a 2-bit priority field is shown, which requires only two logic levels for each magnitude comparator (again, inverters are not shown). The proposed approximate priority magnitude comparator now requires both the greater than and equal outputs to decide whether to deterministically or pseudorandomly route the packet; however, these operate in parallel. Finally, in Figure 8c, the equivalent logic using only a single bit for classifying packet age is shown, leading to a single level of logic.

The above can be used together with the circuit diagram in Figure 7 to estimate the improvement in the critical path timing. The request logic of the permutation block requires two logic levels, the grant logic requires two logic levels, since it is a 3-bit boolean function, as shown from the table in Figure 7, and the two-to-one multiplexer requires two more levels of logic. Therefore, the original permutation block requires a total of eight logic levels and the 2-bit priority field permutation block requires six. The 1-bit priority field is expected to also require six logic levels, since the delay will be dominated by the request logic, which still requires two logic levels and operates in parallel with the magnitude comparator. However, it should provide additional area if not performance gains.

We also expect a reduction in the delay of the selector blocks used in the ejection/injection stage, this time from five logic levels in the original one, to three and two for the 2-bit and 1-bit priority fields, respectively. Since, according to Table 2, the ejection/injection stage accounts for 9.1% of the critical path delay, we can estimate the performance gains by modifying Equation (1) to take this additional analysis into account:

(3) $$tt_{3\mathrm{DHYAPDHYAP}2}=\left(\frac{6}{8}\times \frac{2}{3}\times 71\%+\frac{3}{5}\times 9.1\%+19.9\%\right)\times tt_{3\mathrm{DPERMDPERM}}=60.86\%\times tt_{3\mathrm{DPERMDPERM}}$$

In other words, we expect an additional improvement of 15% from the reduced complexity of each permutation block for a 2-bit priority field. Similarly, for a 1-bit priority field:

(4) $$tt_{3\mathrm{DHYAPDHYAP}3}=\left(\frac{6}{8}\times \frac{2}{3}\times 71\%+\frac{2}{5}\times 9.1\%+19.9\%\right)\times t_{3\mathrm{DPERM}}=59.04\%\times tt_{3\mathrm{DPERMDPERM}}$$

## 4. Experimental Results—High Level Simulation

**Figure 9:**
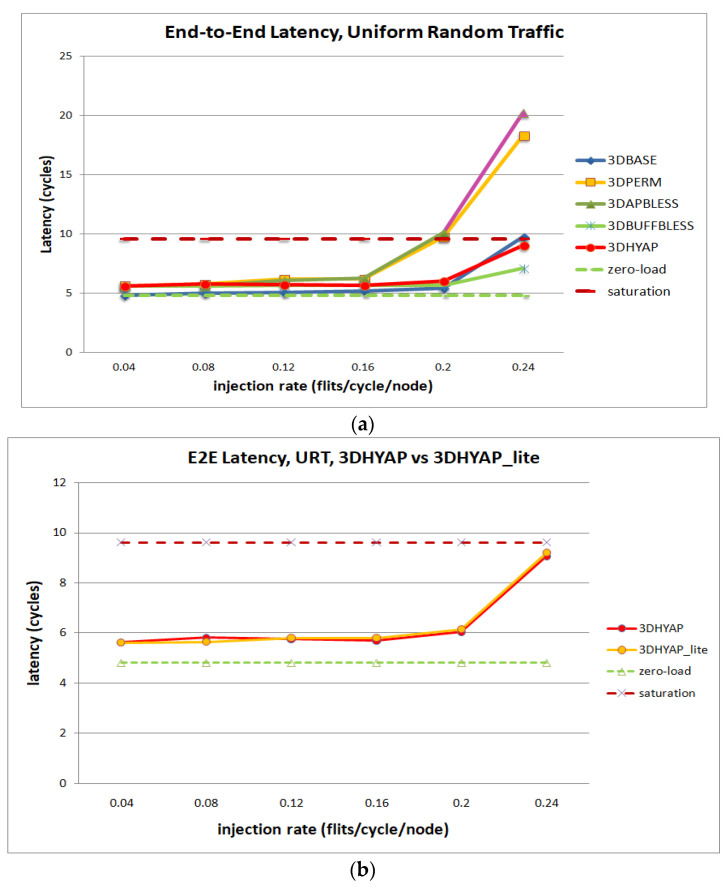
Router latency comparison in cycles for (**a**) uniform random traffic, (**b**) comparison between 3DHYAP and 3DHYAP_lite.

**Figure 10:**
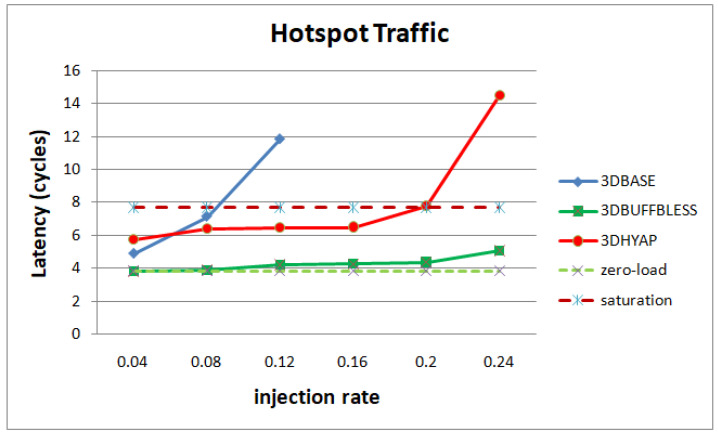
Router latency comparison in cycles for hotspot traffic.

**Figure 11:**
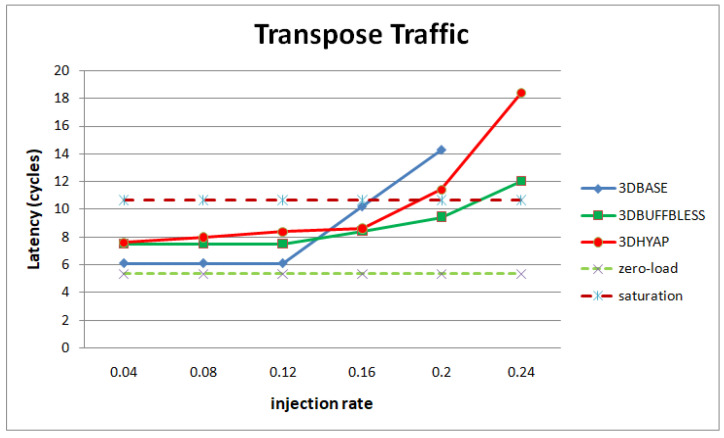
Router latency comparison in cycles for transpose traffic.

**Figure 12:**
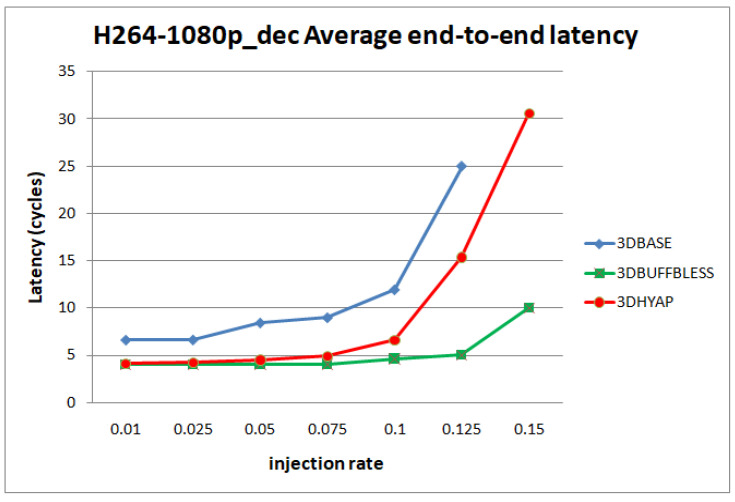
Router latency comparison in cycles for the H.264-1080p video decoder benchmark.

**Figure 13:**
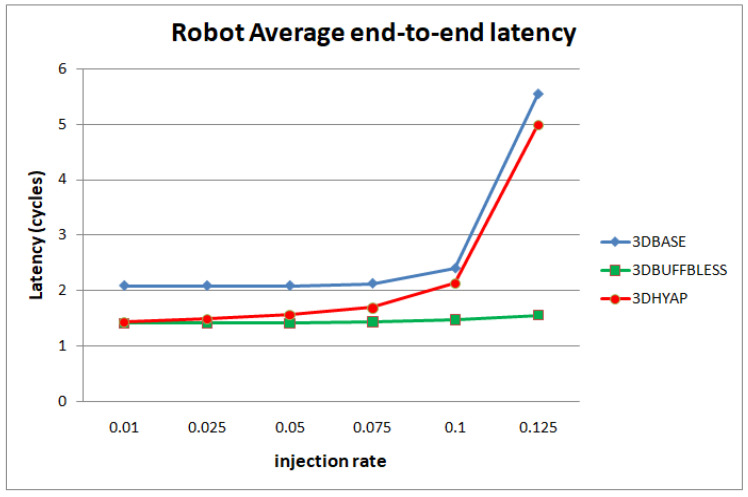
Router latency comparison in cycles for the Robot benchmark.

**Figure 14:**
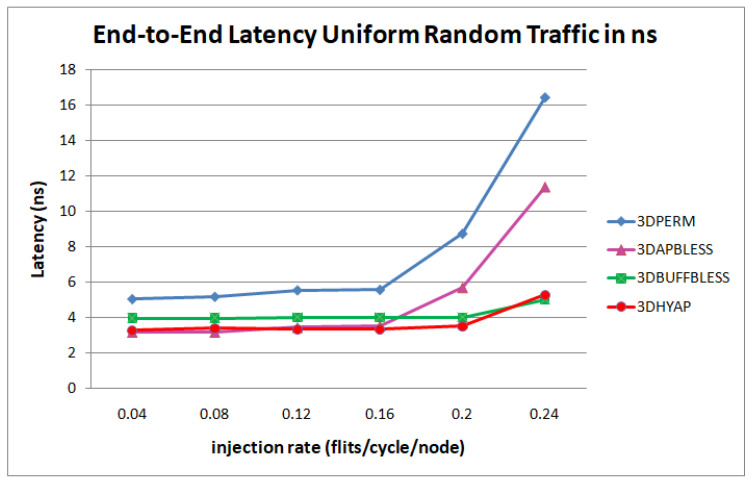
Router latency comparison in ns for uniform random traffic.

**Figure 15:**
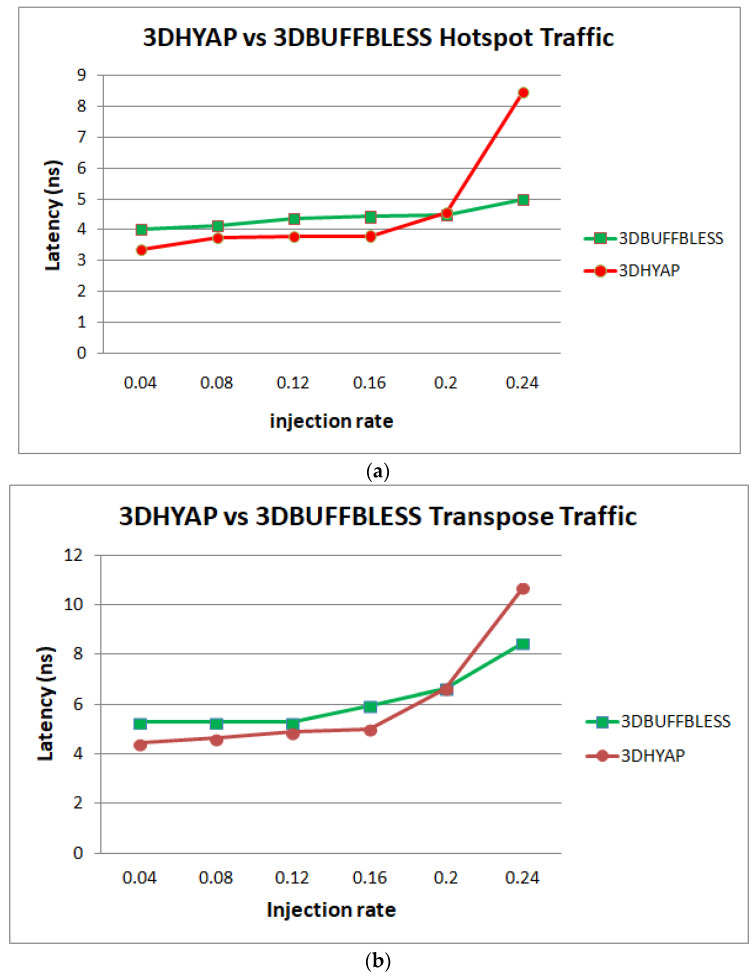
Router latency comparison in ns for (**a**) hotspot and (**b**) transpose traffic.

**Figure 16:**
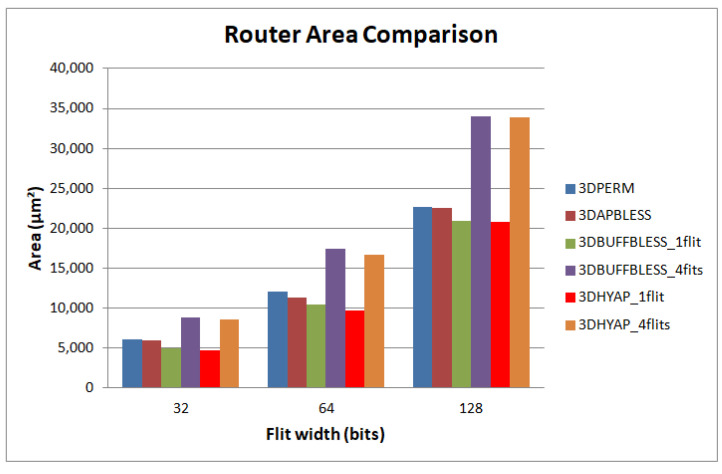
Router area comparison in 45 nm Nangate technology.

For presentation purposes, we divide the evaluation section into the high-level simulation results and the hardware implementation results. The high-level simulations explore the latency in hops under various traffic conditions, while abstracting away irrelevant hardware details, while hardware implementation is used to obtain clock frequency and area and power consumption figures. We then present combined evaluation results that calculate the latency in nanoseconds using a combination of the latency in cycles obtained by high-level simulation and the clock frequency, respectively, obtained from hardware implementation. We considered a single-cycle router in order to achieve a fair comparison between the proposed router and previous work, since many bufferless routers proposed in previous work [15,16] are single-cycle routers, which is not a coincidence, since low latency in NoCs is as important as high throughput, and therefore, deep router pipelines are prohibitive. It would also be unfair to compare single-cycle routers with a pipelined proposed design. Therefore, we implemented all routers as single-cycle, only registering the outputs. Considering a number of N pipeline stages, equal for all routers, then the latency of all routers presented in the following subsections, shown in Figure 8, Figure 9, Figure 10, Figure 11, Figure 12 and Figure 13, would be multiplied by the pipeline stages (assuming no pipelining in the links), and therefore, the figures would be almost identical, scaled by N. Furthermore, in Figure 14, Figure 15 and Figure 16, where the cycle time is taken into account after synthesis, in the case of N pipeline stages, the cycle time would be divided by the pipeline stages (approximately) and the latency in ns would be the same.

The proposed design mainly aims to improve bufferless routing performance while introducing minimal overheads. For that reason, reliability and fault tolerance were not considered. Clearly, methods for addressing transient and permanent faults in both buffered [27] and bufferless routers [28] can also be employed in the proposed router (as well as the previous work evaluated), but that is beyond the scope of the proposed work.

### 4.1. High-Level Simulation Setup

Regarding high-level simulation, we developed cycle-accurate models of 3DBASE, 3DBUFFBLESS, 3DPERM and 3DHYAP in the HNoCs environment [29]. The simulation was performed on a 4 × 4 × 3 NoC mesh. The simulation duration was 4 milliseconds with a warm-up period of 4 microseconds. Synthetic and realistic NoC traffic patterns were implemented to evaluate the performance of the proposed router. In terms of synthetic traffic patterns, we used uniform random traffic, transpose traffic and hotspot traffic, starting with an injection rate of 0.04 flits/cycle/node and stopping at the network saturation point. In uniform random traffic, each source sends to all destinations with equal probability. In hotspot traffic, each source sends to one of the central routers with a probability of 10%, and with an equal probability to the rest. Finally, in transpose traffic, the source router with coordinates (x, y, z) sends to the destination with coordinates (N_x-1-x, N_y-1-y, N_z-1-z), where N_x, N_y, N_z, are the 3D mesh network dimensions.

### 4.2. Simulation Using Synthetic Traffic

Figure 9 illustrates the average end-to-end latency per flit in cycles under Uniform Random Traffic (URF). Specifically, Figure 9a compares 3DBASE, 3DPERM, 3DBUFFBLESS, 3DAPBLESS and 3DHYAP for uniform random traffic. For further clarification, we show the zero-load and saturation latency as defined above. For injection rates less than 0.2 flits per cycle per node, all routers are close to the zero-load latency. It can be seen that the most vulnerable router to saturation is 3DAPBLESS, closely followed by 3DPERM, which begins to saturate at an injection rate of 0.2 flits/cycle/node. These are the bufferless routers using nine permutation blocks, and since 3DAPBLESS misroutes flits more than 3DPERM at high injection rates due to the approximate priority comparison, this result is to be expected.

The next router to begin saturating is 3DBASE at 0.24 flits per cycle per node. This router has very low latency in cycles at low injection rates, since it centrally sorts flits and, therefore, features the fewest deflections among bufferless routers. However, its latency starts rapidly rising after the 0.2 injection point.

3DBUFFBLESS features the lowest saturation overall since it can store some packets instead of deflecting them, but it has a slightly higher zero-load latency. 3DHYAP provides a middle ground between 3DBASE and 3DBUFFBLESS, since it stores some flits like 3DBUFFBLESS, but deflects the remaining flits less efficiently than 3DBASE.

Furthermore, it can be observed that 3DHYAP and 3DBUFFBLESS feature somewhat higher end-to-end latency below 0.2 flits per cycle per node, and significantly lower above. The reason for the higher end-to-end latency in the low injection rates compared to 3DBASE is that some flits spend time stored in the 3DBUFFBLESS buffers, while, in 3DBASE, they are always transmitted in the same cycle. Since the injection rate is low, the deflections are few and that incurs some latency overhead. However, at an injection rate of 0.2 the two routers feature virtually the same latency and, at the higher injection rates, this trend is emphatically reversed with 3DBUFFBLESS featuring significantly lower latency in cycles. This is due to the fact that 3DBASE deflects many flits, while 3DBUFFBLESS can store incoming flits from the up and down ports until a port becomes available, leading to fewer deflections, and thus fewer hops that offset this additional intra-router latency.

3DHYAP and 3DBUFBLESS reach saturation latency at an injection rate of 0.24 hops/flit/node, while the latency in cycles of 3DBASE at the same injection rate is 30% less than that value. 3DBUFBLESS reaches saturation latency at an injection rate of 0.24 hops/flit/node, while the latency in cycles of 3DBASE at the same injection rate is 30% less than that value. Furthermore, as will be discussed in the hardware evaluation results, due to the higher clock frequencies achieved by 3DBUFFBLESS, the gains in latency in ns is approximately 50% of that value.

Figure 9b separately compares 3DHYAP with 3DHYAP_lite (two versus one bit comparison). 3DHYAP_lite shows a slight additional latency compared to 3DHYAP at injection rates above 0.16, since it tends to misroute some flits compared to 3DHYAP, since its priority comparison is less accurate than 3DHYAP.

In Figure 10, we see the same analysis for hotspot traffic. 3DPERM and 3DAPBLESS saturate very rapidly due to many deflections in the central routers, and are not shown. Hotspot traffic is, as expected, more demanding on the network, forcing hops and latency to increase starting from the low injection rates. This has the effect of 3DBASE featuring higher latency than 3DHYAP almost immediately, the only exception being the very low injection rate of 0.04. 3DHYAP eventually reaches the saturation threshold at an injection rate of 0.2 due to many deflections, while 3DBUFFBLESS reaches saturation after 0.24, proving the least vulnerable to saturation.

Figure 11 presents simulation results for transpose traffic. All router average latencies are close to the zero-load latency injection rates below 0.12. However, 3DBASE reaches the saturation threshold at 0.16, while 3DHYAP reaches it at 0.2, which is a relative increase of the saturation threshold by 25% compared to 3DBASE and 3DBUFFBLESS at approximately 0.22. It must be noted that the differences between 3DHYAP and 3DHYAP_lite, as well as 3DHYAP and 3DBUFFBLESS, for buffer sizes above 1 flit, are imperceptible and are not shown in the diagram for simplicity. This is likely because, at low injection rates, they are very close, but when saturation begins they all rapidly saturate.

From the above diagrams, we can generalize that 3DBUFFBLESS and 3DHYAP feature higher zero-load latency than 3DBASE. However, 3DBUFFBLESS degrades much more gracefully as injection rate increases than 3DBASE, with 3DHYAP somewhere in the middle.

### 4.3. Simulation Using Real Traffic Patterns

Real data transmission of NoCs is much less regular than synthetic traffic patterns. In order to capture the performance of the proposed router under these conditions, we implemented the Multi-Constraint System-Level (MCSL) NoC Traffic Patterns proposed in [30] on our 3D design to capture its performance. Due to the long simulation times required, we only compared 3DBASE with 3DHYAP. We used two applications as benchmarks, namely “ROBOT”, which is the Newton-Euler dynamic control calculation for the 6-degrees-of-freedom Stanford manipulator, comprising 88 tasks and 131 communication links, and “H264-1080p_dec”, which is an H.264 video decoder with a resolution of 1080p comprising 5191 tasks and 7781 communication links.

It should be noted that since NoC architectures are used in many applications, from edge applications to big data [31], we intend the proposed router to be a general-purpose router for 3D NoCs. Therefore, the above benchmarks were only used for evaluation and the proposed architecture was only designed with the limitations of 3D integration in mind and not a specific application. A specific application would likely afford additional optimization in the router, or at the network level; for example, different buffering requirements for the up direction than the down direction, or a preference for deflection, etc.

Figure 12 and Figure 13 show the average end-to-end latency as a function of the injection rate for the H.264 video decoder application and the Robot application, respectively. In general, the trends observed using synthetic traffic patterns are present here too: 3DBASE reaches saturation first, followed by 3DHYAP, with 3DBUFFBLESS being the last to saturate. One pronounced difference is that 3DBASE features higher latency in cycles from the start, even at very low injection rates.

## 5. Experimental Results—Hardware Evaluation

In this section, we show and extensively discuss the implementation results from synthesizing the proposed router and its counterparts in the Nangate 45 nm library [32]. Then, we combine the hardware performance with the simulations of the previous section to obtain latency in nanoseconds for each router, instead of cycles.

### 5.1. Performance Evaluation

Table 4 compares 3DHYAP with 3DPERM, 3DBUFFBLESS and 3DAPBLESS in terms of maximum operating frequency in GHz for flit widths of 32, 64 and 128 bits.

From the above table, it can be seen that 45 nm implementation results generally agree with the results of the analysis in Section 3.1. Specifically, 3DHYAP achieves an improvement of about 48% in terms of maximum operating frequency compared to 3DPERM, and 20% compared to 3DBUFFBLESS, depending on flit size. These results are consistent with the predictions of Equations (1), (3) and (4).

Figure 14 and Figure 15 revisit the simulation results in Section 4.2, Figure 9, Figure 10 and Figure 11, but the latency is given in ns after multiplying the cycles of each router by its clock period corresponding to the operating frequencies of Table 4.

Figure 14 shows that 3DHYAP outperforms the other routers until the injection rate of 0.24 flits per cycle per node, where saturation begins. This includes the state-of-the-art bufferless routers 3DPERM and 3DAPBLESS, as well as the partially buffered 3DBUFFBLESS. In particular, 3DHYAP features an average latency approximately half that of 3DPERM, with only one third of the latency of 3DPERM at an injection rate of 0.24. Compared to 3DAPBLESS, the proposed router is very close in latency at the low injection rate, but 3DAPBLESS begins to rapidly saturate above 0.16 flits/cycle/node, while 3DHYAP starts exhibiting signs of saturation at 0.24 flits/cycle/node, where it features less than half the average latency of 3DAPBLESS. Finally, compared to 3DBUFFBLESS, which is also partially buffered, 3DHYAP exhibits a latency reduction of about 17% before saturation begins. This latency reduction is due to the higher clock frequency achieved by 3DHYAP due to the approximate deflection mechanism.

Similarly, Figure 15 shows that 3DHYAP outperforms 3DBUFFBLESS until the injection rate of 0.2 flits per cycle per node, where saturation begins. In other words, when clock period is taken into account, 3DHYAP provides the lowest latency as predicted.

### 5.2. Area Evaluation

Figure 16 compares routers in terms of area in a 45 nm technology. The proposed router is demonstrated to be the most area efficient of all designs. The analysis which led to Equation (2) predicted an area reduction compared to bufferless routing of 20%, and this is confirmed by the experimental results of Figure 16 for a buffering of one flit. It can be seen that, with a buffer size of one flit, 3DBUFFBLESS is more area efficient than 3DPERM, achieving an area reduction of 20%, roughly in accord with Equation (2).

Compared to 3DBUFFBLESS, which is also buffered in a similar way, the proposed router demonstrates a small area reduction due to the approximate priority comparison, leading to the simpler permutation logic of Figure 8. Increasing the buffer size to two flits offsets the gains of using a smaller permutation network due to the size of the buffers, which are not taken into account in Equation (2), while a buffer size of four flits increases this even more dramatically, leading to an increase of area of 25% and 40%, respectively.

However, our simulations have shown that even with a buffer size of one flit, 3DHYAP outperforms 3DBUFFBLESS and 3DPERM in terms of latency; while increasing the buffer size does not significantly add benefit, the bufferless ports dominated the buffered ones when saturation begins. This causes the network to saturate at approximately the same injection rate, independent of buffer size. Therefore, the minimum buffering of one flit is preferred, since there is no reason to offset the area gains and impose an area overhead with no performance gains.

### 5.3. Power Consumption Evaluation

**Figure 17:**
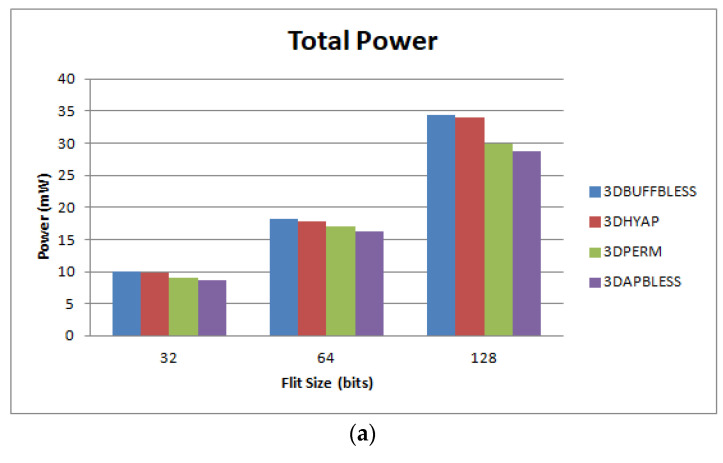

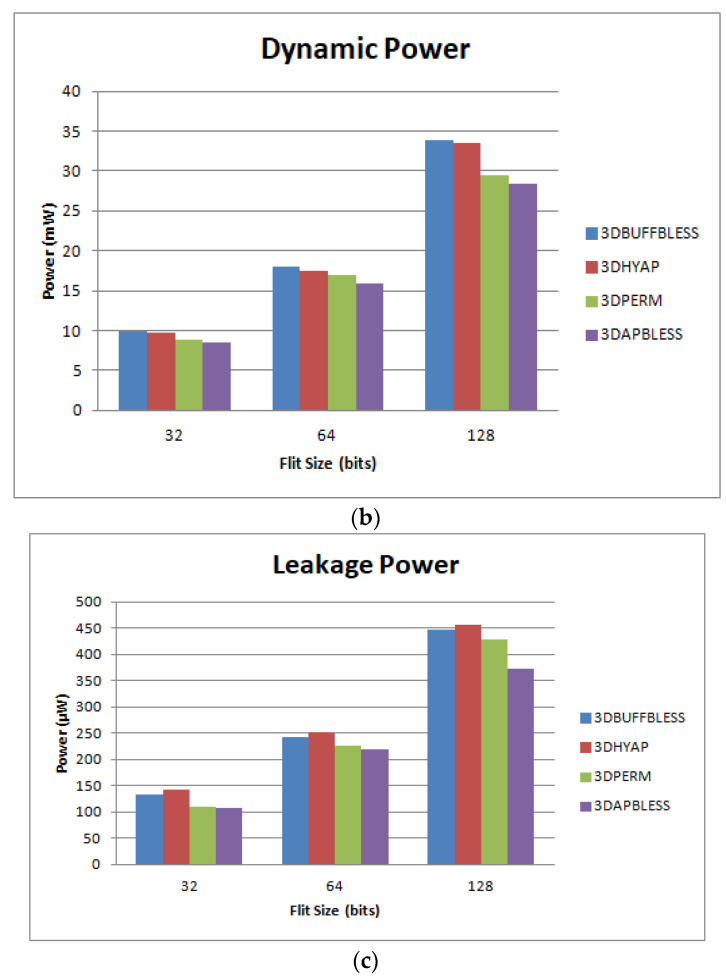
Router power consumption comparison. Total power (**a**), dynamic power (**b**) and leakage power (**c**).

We measured the power consumption of the proposed router and its counterparts for flit sizes of 16, 32 and 64 bits at a clock frequency of 1 GHz in the same 45 nm technology. The results are shown in Figure 17. Figure 17a shows total power consumption, while Figure 17b shows dynamic power consumption and Figure 17c shows leakage power. It can be seen that, as expected, the bufferless routers consume about 15% less power than the buffered ones. It is also evident that, at the target 45 nm technology, power consumption is heavily dominated by dynamic power, since the Figure 17a and Figure 17b are almost identical. Regarding leakage power, the completely bufferless routers are again shown to be more efficient, with the proposed router and 3DBUFFBLESS consuming about 20% more leakage power, depending on flit size.

**Figure 18:**
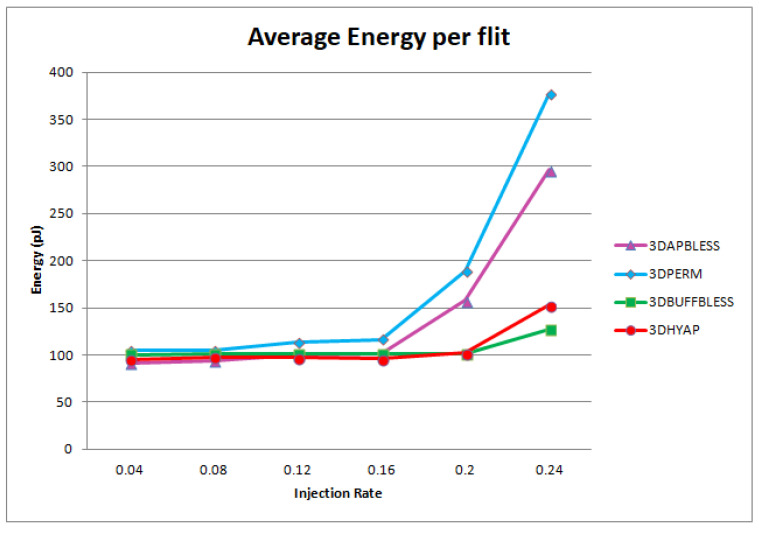
Router energy efficiency comparison for uniform random traffic.

However, a different picture emerges when considering the total energy efficiency. Figure 18 combines the results of Figure 14 and power consumption to demonstrate the average energy required per flit transmission for uniform random traffic. Results are shown for 64-bit flits. The results are similar for 32- and 128-bit flit sizes, since the energy per flit is shown to be almost constant (actually slightly decreasing with flit size for all routers). As shown in Figure 18, at the low injection rates, all routers approximately require the same energy to send a flit, but, as the injection rate rises, and as bufferless routers saturate earlier, they also require more energy to send a flit, despite being more power efficient. Therefore, the power overhead imposed by 3DBUFFBLESS and 3DHYAP shown in Figure 17 translates to energy efficiency due to more efficient routing.

## 6. Conclusions

This paper presented an exploration of combining partially buffered routing in the z dimension of a 3D router, with approximate priority deflection routing in the x and y dimensions. From the combination of high-level simulation with hardware implementation, the key results summarized below were obtained:

Firstly, minimal buffering in the z dimension significantly increases the saturation threshold in a 3D mesh topology compared to completely bufferless routing. However, somewhat counterintuitively, additional buffering has minimal effect, as also demonstrated in [17]. The lower latency is also translated to higher energy efficiency, despite the power overhead imposed by the partial buffering.

Secondly, the reduction of the bufferless routing to four ports instead of six significantly reduces the critical path delay, and therefore, increases the clock frequency.

Thirdly, adding an approximate priority comparison further increases clock frequency and reduces router area at the expense of somewhat lower saturation latency.

Finally, the zero-load latency of the partially buffered routers is somewhat higher than the bufferless ones due to the buffered ports. However, this is likely to be improved by adding pipeline stages, which are left for exploration in the future.

Further considering possible future research directions, we plan to evaluate the proposed router, as well as the counterparts discussed in the paper, in terms of reliability and fault-tolerance using gate-level reliability estimation tools, such as those proposed in [33]. In fact, to the best of our knowledge, such an analysis and comparison between buffered and bufferless routers has not yet been attempted.

## Figures and Tables

**Table 1:** Bufferless router area breakdown.

Sub-Module	Area (%)
Permutation network	57.5%
Ejection/injection stage	35.6%
Header updater	2.7%
Other	4.2%

**Table 2:** Bufferless router critical path breakdown.

Sub-Module	Delay (%) of Critical Path
Permutation network	71%
Ejection/injection stage	9.1%
Header updater	7.5%
Port request logic/Other	12.38%

**Table 3:** 3DHYAP priority classes.

Priority Class	2-Bit Priority Field	1-Bit Priority Field
young	00	0
fairly young	01	
fairly old	10	
old	11	1

**Table 4:** Maximum Frequency Comparison.

Flit Size (Bits)	Maximum Operating Frequency (GHz)			
	3DPERM	3DBUFFBLESS	3DAPBLESS	3DHYAP
32	1.115	1.412	1.781	1.710
64	1.114	1.391	1.650	1.619
128	1.100	1.371	1.638	1.607

## Data Availability

Data is contained within the article.

## Abbreviations

NoC	Network-on-Chip
URT	Uniform Random Traffic
ASIC	Application-Specific Integrated Circuit
PSRNG	Pseudo-Random Number Generator

## References

[1] Tatas K., Siozios K., Soudris D., Jantch A. (2014). Designing 2D and 3D Network-on-Chip Architectures.

[2] Wang L., Ma S., Li C., Chen W., Wang Z. (2017). A High Performance Reliable NoC Router. Integration.

[3] Benini L., De Micheli G. (2006). Networks on Chips: Technology and Tools.

[4] Jafari F., Lu Z., Jantsch A., Yaghmaee M.H. Optimal regulation of traffic flows in network-on-chip. Proceedings of the Conference on Design, Automation and Test in Europe (DATE).

[5] Jafari F., Lu Z., Jantsch A., Yaghmaee M.H. (2010). Buffer Optimization in Network-on-Chip Through Flow Regulation. IEEE Trans. Comput. Aided Des. Integr. Circuits Syst..

[6] Park J., O’Krafka B.W., Vassiliadis S., Delgado-Frias J. Design and evaluation of a DAMQ multiprocessor network with self-compacting buffers. Proceedings of the 1994 ACM/IEEE Conference on Supercomputing.

[7] Nicopoulos C.A., Park D., Kim J., Vijaykrishnan N., Yousif M.S., Das C.R. ViChaR: A dynamic virtual channel regulator for network-on-chip routers. Proceedings of the 9th Annual International Symposium on Microarchitecture (MICRO).

[8] Ramanujam R., Soteriou V., Lin B., Peh L.S. Design of a High-Throughput Distributed Shared-Buffer NoC Router. Proceedings of the 4th ACM/IEEE International Symposium on Networks-on-Chip.

[9] Khan K., Pasricha S., Kim R.G. RACE: A Reinforcement Learning Framework for Improved Adaptive Control of NoC Channel Buffers. Proceedings of the Great Lakes Symposium on VLSI 2022.

[10] Fang J., Mao Y., Cai M., Zhao L., Chen H., Xiang W. (2022). STTAR: A Traffic- and Thermal-Aware Adaptive Routing for 3D Network-on-Chip Systems. Comput. Mater. Contin..

[11] Kodi A., Louri A., Wang J. Design of energy-efficient channel buffers with router bypassing for network-on-chips (NoCs). Proceedings of the 10th International Symposium on Quality Electronic Design.

[12] Moscibroda T., Mutlu O. A Case for Bufferless Routing in On-Chip Networks. Proceedings of the 36th Annual International Symposium on Computer Architecture.

[13] Fallin C., Craik C., Mutlu O. Chipper: A low-complexity bufferless deflection router. Proceedings of the 17th IEEE International Symposium on High Performance Computer Architecture.

[14] Michelogiannakis G., Sanchez D., Dally W.J., Kozyrakis C. Evaluating Bufferless Flow Control for On-Chip Networks. Proceedings of the 4th ACM/IEEE International Symposium on Networks-on-Chip (NOCS 2010).

[15] Yao C., Feng C., Zhang M., Wei S. DualBLESS: Bufferless Router with Dual Ejection Ports for 2D and 3D NoC. Proceedings of the International Conference on Engineering Technology and Application (ICETA 2015).

[16] Feng C., Lu Z., Jantsch A., Zhang M. (2012). A 1-Cycle 1.25 GHz Bufferless Router for 3D Network-on-Chip. IEICE Trans. Inf. Syst..

[17] Tatas K., Savva S., Kyriacou C. 3DBUFFBLESS: A Novel Buffered-Bufferless Hybrid Router for 3D Networks-on-Chip. Proceedings of the 27th International Symposium on Power and Timing Modeling, Optimization and Simulation (PATMOS 2017).

[18] Kunthara R.G., James R.K., Sleeba S.Z., Jose J. Asymmetric routing in 3D NoC using interleaved edge routers. Proceedings of the 12th International Workshop on Network on Chip Architectures (NoCArc 2019).

[19] Sujata S.B., Sandi A.M. (2022). Design and analysis of buffer and bufferless routing based NoC for high throughput and low latency communication on FPGA. Int. J. Pervasive Comput. Commun..

[20] Tatas K. High-performance 3D NoC bufferless router with approximate priority comparison. Proceedings of the 7th International Conference on Modern Circuits and Systems Technologies (MOCAST).

[21] Wang L., Wang X., Wang Y. (2019). An Approximate Bufferless Network-on-Chip. IEEE Access.

[22] Hayenga M., Jerger N.E., Lipasti M. Scarab: A single cycle adaptive routing and bufferless network. Proceedings of the 42nd Annual IEEE/ACM International Symposium on Microarchitecture (MICRO 2009).

[23] Jiang X., Zeng L., Watanabe T. (2014). A Sophisticated Routing Algorithm in 3D NoC with Fixed TSVs for Low Energy and Latency. IPSJ Trans. Syst. LSI Des. Methodol..

[24] Arka A.I., Gopal S., Doppa J.R., Heo D., Pande P.P. (2020). Making a Case for Partially Connected 3D NoC: NFIC versus TSV. ACM J. Emerg. Technol. Comput. Syst..

[25] Matsutani H., Bogdan P., Marculescu R., Take Y., Sasaki D., Zhang H., Koibuchi M., Kuroda T., Amano H. A case for wireless 3D NoCs for CMPs. Proceedings of the IEEE 18th Asia and South Pacific Design Automation Conference (ASP-DAC 2013).

[26] Matsutani H., Koibuchi M., Fujiwara I., Kagami T., Take Y., Kuroda T., Bogdan P., Marculescu R., Amano H. Low-latency wireless 3D NoCs via randomized shortcut chips. Proceedings of the IEEE Design, Automation and Test in Europe Conference and Exhibition (DATE 2014).

[27] Shafique M.A., Baloch N.K., Baig M.I., Hussain F., Zikria Y.B., Kim S.W. (2020). NoCGuard: A Reliable Network-on-Chip Router Architecture. Electronics.

[28] Feng C., Lu Z., Jantsch A., Zhang M., Xing Z. (2013). Addressing Transient and Permanent Faults in NoC with Efficient Fault-Tolerant Deflection Router. IEEE Trans. Very Large Scale Integr. (VLSI) Syst..

[29] Ben-Itzhak Y., Zahavi E., Cidon I., Kolodny A. HNOCS: Modular open-source simulator for Heterogeneous NoCs. Proceedings of the International Conference on Embedded Computer Systems (SAMOS).

[30] Liu W., Xu J., Wu X., Ye Y., Wang X., Zhang W., Nikdast M., Wang Z. A NoC Traffic Suite Based on Real Applications. Proceedings of the IEEE Computer Society Annual Symposium on VLSI (ISVLSI).

[31] Fang J., Liu S., Liu S., Cheng Y., Yu L. (2018). Hybrid Network-on-Chip: An Application-Aware Framework for Big Data. Complexity.

[32] 15 nm Open-Cell Library and 45 nm FreePDK. https://si2.org/open-cell-library/.

[33] Pontes M.F., Farias C.R., Schvittz R.B., Butzen P.F., Leomar S.R. (2021). Survey on Reliability Estimation in Digital Circuits. J. Integr. Circuits Syst..

